# Supplementary material for: Geospatial distribution and predictors of postnatal care utilization during the critical time in Ethiopia using EDHS 2019: A spatial and geographical weighted regression analysis
Source: PLoS One. 2024 Dec 30;19(12):e0309929. doi: 10.1371/journal.pone.0309929 (PMC11684686; doi:10.1371/journal.pone.0309929)
Supplement: Attachment — Submitted filename: respose to reviewers.pdf [file pone.0309929.s002.pdf]

Spatial distribution and application of geographical weighted regression analysis to assess the predictors of postnatal care utilization during the critical time hot spots in Ethiopia using EDHS 2019

Muluken Chanie Agimas[1], Tigabu Kidie Tesfie[1], Nebiyu Mekonnen Derseh[1], Meron Asmamaw[1], Habtamu Wagnew Abuhay[1], Getaneh Awoke Yismaw[1]

[1]Department of Epidemiology and Biostatistics, institute of public health, college of medicine, university of Gondar, Gondar, Ethiopia.

1. Muluken Chanie Agimas (MCA): mulukensrc12@gmail.com
2. Tigabu Kidie Tesfie (TKT): tigabukidie@gmail.com
3. Nebiyu Mekonnen Derseh (NMD): nebiyumek12@gmail.com
4. Meron Asmamaw (MA): merryalem101@gmail.com
5. Habtamu Wagnew Abuhay (HWA): hab7wagnew@gmail.com
6. Getaneh Awoke Yismaw (GAY): getanehawoke9@gmail.com

Corresponding author:

(MCA): mulukensrc12@gmail.com

To: PLOS ONE <em@editorialmanager.com>

Manuscript Number: PONE-D-23-41347

Cover Letter for reviewer's comments and authors responses

We would like to thank both the Editorial board members and reviewers for kind comments and suggestions. Accordingly, we have tried to correct and respond to those comments point by point, page by page, and line by line. Therefore, we prepared this author's response, the revised manuscript, the separate revised figures and tables, and the tracked changes of the manuscript.

Sincerely, with best regards!

Muluken Chanie Agimas

ID: https://orcid.org/0000-0003-0808-124X

**Comments from review and editorial board**	**Authors response**
**Editor's comment**	
Your ethics statement should only appear in the Methods section of your manuscript.	Dear Editor, It is a very interesting comment, and we accepted it so that we can assure you that an amendment has been made.
We note that your Data Availability Statement is currently as follows: All relevant	Thank you, dear editor. We were restricted from sharing the data set without permission from DHS International. So we

data are within the manuscript and its Supporting Information files.<br><br>Please confirm at this time whether or not your submission contains all raw data required to replicate the results of your study. Authors must share the "minimal data set" for their submission.	try to amend the availability statement to say, "All relevant data are within the manuscript."
**Reviewer-1 comments**	Authors response
**General Questions:**<br><br>Is it really necessary to conduct additional research when a comparable study was completed in Ethiopia? What will it add to the already known facts? What was the gap in the previous study? Why you select geographical weighted regression analysis for this study?	Dear reviewer, It is a very interesting comment. We are interested in using weighted regression because geographically weighted regression (GWR) allows relationships in a regression model to vary over space. In contrast to traditional linear regression models, which have constant regression coefficients over space, regression coefficients are estimated locally at spatially referenced data points with GWR. Additionally, the motivation for the use of GWR is to consider spatially varying relationships between covariates and an outcome variable. The multilevel analysis cannot consider or capture spatially varying relationships between covariates and an outcome variable. To apply specific interventions to a specific geographical location and resource allocation, the effect of predictors across the geographical area should be assessed. The effect of each predictor may vary across the geographical areas of Ethiopia because of culture, socioeconomic differences, health-seeking behaviour, and so on. The multilevel analysis does

	not consider this issue. Previous studies did not examine the effect of each predictor across geographical areas. Additionally, we are interested in conducting this study because, in Ethiopia, there has been no research conducted on early PNC for evidence-based health interventions using the recent EDHS 2019 data set.<br><br>**to explain more:**<br>The majority of the previous studies were focused on factors associated with PNC in general, whereas studies on PNC utilisation during the critical period were not adequate. Even though one national-level study was conducted on early PNC in Ethiopia using EDHS 2016 data and another local-level study was conducted in Sidama, Ethiopia**, i**t did not allow us to know about the effect of each predictor across the geographical areas of Ethiopia. Because all possible factors have no equal impact on PNC utilisation during the critical time across the regions, Additionally, the spatial distribution of PNC utilization during the critical time is not equally distributed across the corner of Ethiopia. So regional-level estimates of PNC utilization during the critical time are highly important to inform policymakers, planners, and other

	stakeholders about the population where the burden is high and fair resource allocation. Previous studies were not used for this purpose. According to our knowledge and searching, in Ethiopia, there is no study or evidence to show the spatial distribution and application of geographical weighted regression to show the effect of each predictor across the geographic areas of Ethiopia. Therefore, the current study aimed to assess the spatial distribution and application of geographical weighted regression analysis to identify the predictors of PNC utilization during the critical time hot spots in Ethiopia using EDHS 2019.
Since EMDHS 2019 the data were not collected all variable like the variables; Media exposure such as (internet use, watching TV, listening radio, and reading newspaper), husband education status, maternal working status and so on. These variables were the most significant factor for postnatal utilization during the critical time.	Dear reviewer, You are right; the most important variables may be missed in the secondary data (EDHS). We cannot get all the variables we are interested in analyzing. We try to state this issue as a limitation of the study and as a recommendation for future researchers to include such variables using the primary data collection method. But regarding the media exposure, it is collected in the EDHS 2019. Thus, we use this variable by defining it as follows: Media exposure was assessed based on whether people had access to read newsletters, listen to the radio, and watch TV. Accordingly, if they have access to all three media (newsletter, radio, and

	TV) at least once a week, we categorized them as "yes," otherwise "no.”
In abstract section particularly in result and conclusion part, it is too lengthy & try to summarize the sentence and it should incorporate the method that you used for spatial analysis.<br>Study design and setting section you should study area map. Since your study, analysis was spatial regression so it is better to show your study area within a single map.	Dear reviewer Thank you so much for your excellent view. You are right; the result and conclusion parts are very detailed. Therefore, the necessary amendment has been made accordingly. Regarding the spatial analysis, we try to incorporate all methods of analysis. Examples include SAT scan analysis, autocorrelation, and hot spot analysis methods. We also include the area map of Ethiopia as an amendment in the study setting.
In your Sampling procedure and sampling technique section, “2105 women aged 15-49 years who give birth preceding 2019 EDHS survey were included for the study”. It is weighted sample size or not? It should be clear it maybe weighted or unweighted sample size.<br>In your method section especially spatial analysis part, you should write all spatial analysis technique separately like spatial auto-correlation, hotspot analysis, and interpolation and SaTScan analysis and cite appropriate citation. In addition, why you select ordinary kriging interpolation technique from other interpolation technique like deterministic like inverse distance weighted(IDW) and geostatistical interpolation	Dear reviewer, We really appreciate your detailed view. We have seen our manuscripts again, one by one. We have used the weighted sample size to be more representative. Regarding the separate write-up of spatial analysis techniques, we have done so based on what you have commented. We used the ordinary Kriging interpolation technique because ordinary Kriging has the advantages of being the best linear unbiased estimator and accounts for clustering and screening effects. The main advantages of kriging include:<br><br>1. **Rigorous statistical framework:** Kriging is a rigorous statistical method that incorporates a measure of uncertainty and provides confidence intervals for the estimated values.

methods among those methods simple, ordinary Kriging and universal Kriging?	2. **Optimal interpolation:** Kriging provides the optimal linear unbiased estimate of the variable of interest, given the assumptions about the data distribution and spatial autocorrelation.<br>3. **Ability to incorporate auxiliary data:** Kriging can incorporate auxiliary data such as covariates or secondary data sources to improve the accuracy of the estimates.<br>4. **Flexibility:** Kriging can be applied to various types of data, including continuous, categorical, and count data.
Note: See the results section, especially table 3 for the OLS result. Since your explanatory variables, such as primary education and delivery by CS, were not statically significant variables, your OLS result is unreliable and incorrect. As a result, you should reanalyze your spatial regression by removing those two variables in OLS and GWR analysis and map out all statically significant variables. This breaks the assumption of OLS analysis that violet explanatory variables are statically significant. Rewrite your abstract, results, discussion, and conclusions section overall in light of the updated data.	Dear reviewer, we are really surprised to see such a report. Thank you so much for your detailed view and your contribution to this valuable paper. So based on your comment, we reanalyzed it again. The results showed that the three variables like ANC visit, facility delivery, and media exposure are still significant, but their beta coefficients have changed. Another additional variable, "no education," is significant, but primary education and delivery by CS were not significant. Both AIC, R^2, and other statistics were changed when we reanalyzed. Based on these findings, the revision has been made both in the result, discussion, abstracts, and conclusion parts.
Reviewer two	Authors response

Introduction: The introduction in the third line states the death rate which does indicate what death. In the next line, it expresses maternal and child mortality. Thus, can you please write the idea sequentially? The other next statement shows the highest death but not much attention was given. Even considering the research published maternal and child issues account for more than 80% of all articles. If considering several NGOs, government programs, and other aids the issue related to maternal and child health takes the greater portion. Thus, can rewrite the whole abstract in a more plausible scientific way of writing?	Dear reviewer It is great. In the third line, the word "death" implies both maternal and new-born deaths. Dear reviewer, we hope that we try to explain the burden of maternal and newborn deaths, then we try to explain the solution for this problem and the gap like this. "**Sub-Saharan Africa contributes the highest maternal and newborn deaths rate. To reverse this problem early postnatal care is the best strategy but there is no study to show the spatial distribution and application of geographical weighted regression to show the effect of each predictor on early postnatal care across the geographic areas in Ethiopia using the recent EDHS 2019 data."**<br>Thus, we hope that the idea is sequential and written in an appropriate way.
Please follow the following more common method of writing an abstract:<br>The first two lines must	Dear reviewer Thank you so much for your comment again. You are right. The way of writing the abstract that you suggest is one of the options. But also, different research books, articles, and scholars are also recommended, like this:

encompass the context of the study and the research problem, further two lines must be covered the objective of the papers with unfolding the description of the title. In the next 2 to 4 lines the methodology will be covered. Afterward, the next two lines are for result and performance. In these lines, the author must define how the results and performance are being achieved, for instance, by conducting either simulation or physical implementation. Please mention the name of the simulation or the physical method. The result statistics must be mentioned in the last two lines and either in percentage or with real-time values.- Objective should be clear and precise.	**In the introduction of the abstract:**<br>1. First define the problem<br>2. Show the global and local burden on of the problem<br>3. The solution of the problem<br>**4.** The gap of the previous study.<br>**In the method of abstract:**<br>1. Design<br>2. Sample size<br>3. Analysis techniques<br>4. Significance test.<br>**In the result section:**<br>1. Main findings example prevalence of early PNC, and factors associated with early PNC, hot spot areas……<br>**In the conclusions of abstract:**<br>1. Summary of the finding based on the main finding.<br>**Dear reviewer, therefore, our abstract was based on the above basic guidelines.**

Method: The method mentions the source of data but does not any method of how the data is handled (extracted, missing handled…etc).<br>The result section even misses the major finding like in proportion or whatever in the very first line.	Dear reviewer Thank you so much for your interesting comments. You are right, we missed such important information (missing data management, source of data, and proportion of PNC during the critical time). Now we amend it accordingly.
Conclusion: The conclusion is comprehensive but could be more specific about the optimal PNC and what to do to improve because you just said to improve PNC. Thus, be more practical in both conclusions.	Dear reviewer, we describe how to improve PNC during the critical time like this: "Expanding antenatal care, facility delivery, education, and media access are highly recommended to improve the utilization of PNC service during the critical time in Ethiopia."
Introduction<br>- The introduction could be clearer and more concise but still contain more important facts that could clearly show the gap in the literature.<br>- It provides less extensive background information about PNC and child and its potential impact on maternal health. It	Dear reviewer, Our study was not on the overall PNC. There have been a lot of studies conducted on PNC and its associated factors. But this study was about PNC during the critical time. Because at this time, both maternal and newborn deaths are higher than at other times. The majority of the previous studies were focused on factors associated with PNC, and studies on PNC utilisation during the critical period are not adequate. Even though one national-level study was

might be more effective if the text is more concise and focused on the most crucial information, including clearly identifying the gap in knowledge that this study is addressing. Many studies already discussed this information and it is already overwhelming, if not only for using geographically weighted regression. Reorganizing ideas could be very important and language editing might be also crucial.	conducted on early PNC in Ethiopia using EDHS 2016 data, it did not allow us to know about the effect of each predictor across the geographical areas of Ethiopia. Because all possible factors have no equal impact across the regions, even the prevalence of early PNC utilization is not equally distributed across the country. Additionally, the previous study did not show the spatial distribution of PNC during the critical time. So regional-level estimates of early PNC are highly important to inform policymakers, planners, and other stakeholders about the population where the burden is high and fair resource allocation. Previous studies were not used for this purpose. According to our knowledge and searching, in Ethiopia, there is no study or evidence to show the spatial distribution and application of geographical weighted regression to show the effect of each predictor across the geographic areas of Ethiopia. That is why we aimed to assess the spatial distribution and application of geographical weighted regression analysis to identify the predictors of PNC utilization during the critical time hot spots in Ethiopia using EDHS 2019.

	Regarding the impact of PNC utilisation during the critical time on maternal health, we have stated in lines 84–86 how to improve maternal health, and it stated like this:<br><br>“The majority of deaths are overcome through immediate PNC service. Just using PNC utilisation based on the recommended time, 60,000 newborn deaths can be reduced, and in Ethiopia, it can be reduced by 10–27%.“<br><br>Dear reviewer, regarding the idea flow of the introduction, we have now made an amendment in a more structured way.<br>Regarding the knowledge gap from the previous studies, we described it in lines 89 to 101.
The method section could include a brief note on<br>- Data Preparation<br>- Brief description of population, sampling, and how the final sampling arrived on<br>- Description and definition of included variables in table form<br>- The inclusion and exclusion criteria are well outlined, but the section could be more concise. For easier readability, the extensive list could be presented in a more summarized and	Dear reviewer, Thank you so much for the invitation to revise the method section. Based on your recommendation, the necessary amendment has been made. Regarding the data preparation, we have already discussed it in lines 158–161. Thus, data were prepared before analysis by cleaning, recoding, missing data management, and weighting the sample.<br>Dear reviewer, regarding the advantages of the selected method, we have stated the advantages of each method we have used. Example: We try to explain why we used the ordinary kriging method, GWR, Sat scan analysis,

organized format, possibly in a table or listed format.<br>- Brief description of the selected method and its advantages over another method i.e. reason for picking it. Those sections should be under specific subheadings.	multistage sampling method, global Moran's I statistics, etc. ……
- The source of data was mentioned but there is no clear guide other people can access and conduct other studies or there is the link to access the datasets. The link you provided is not the right link. It is the link to the page not to data. Just refer to this, it is available in the resource used in this dataset.	Dear reviewer here is the link of the dataset of EDHS 2019. https://www.dhsprogram.com/data/dataset/Ethiopia_Interim-DHS_2019.cfm?flag=1
- Explanation regarding the possibility of bias and Multicollinearity needs addressed	Dear reviewer, regarding Multicollinearity, it is written in the methodology section at lines 199-201. The possible bias has been Witten in line 129.
Result<br>This section look well write, but could benefited from more editing of language, combination of numbers and texts.	Okay thank you. We revised accordingly.
Discussion<br>- The section could provide a more in-depth comparison with existing literature, especially contrasting findings and	Dear reviewer, For a more detailed discussion, we have now compared our findings with many relevant pieces of literature. Additionally, we added the contribution of the findings to the body of knowledge.

methodologies. Further, discussing how this study adds to the existing body of knowledge could be more explicit<br>- The limitations section should be expanded to include limitations related to the methodology used, data, sampling, representatively, inclusion criteria, or analysis methods.	Dear reviewer, we try to list the limitations of the study at the end of the discussion like this: **But it has limitations, such as the fact that the current study uses the geographic coordinates of clusters (2 kilometers for urban areas and 5 kilometers for most clusters in rural areas), making it difficult to estimate the cluster effect in the spatial analysis. Secondly, the current study used secondary data, and thus, additional important public health variables were not included.**
Conclusion:<br>- The main conclusions of the study should be presented more clearly and concisely. It's essential to summarize the key findings and their implications straightforwardly to ensure that readers can easily grasp the main takeaways from the research.<br>- The conclusion should include more detailed recommendations for future research, specifically addressing the gaps and limitations identified in the review. Clear and actionable suggestions for future research would enhance the usefulness of the conclusion<br>- The conclusion could elaborate more on the policy implications of the findings. How should policymakers approach<br>- Provide more explicit	Dear Editor, it is great! You are right. The points you suggest being added to the conclusion may make the conclusion more detailed (not concise). If it is not in more detail, a necessary amendment has been made now.

conclusions regarding the importance of considering contextual factors such as socio-demographic characteristics, women’s empowerment, and partner support	

Thank you, dear editor and reviewers, for your great scientific contribution!
