# Supplementary material for: Geospatial distribution and predictors of postnatal care utilization during the critical time in Ethiopia using EDHS 2019: A spatial and geographical weighted regression analysis
Source: PLoS One. 2024 Dec 30;19(12):e0309929. doi: 10.1371/journal.pone.0309929 (PMC11684686; doi:10.1371/journal.pone.0309929)
Supplement: Attachment — Submitted filename: respose to reviewers.pdf [file pone.0309929.s004.pdf]

# Geospatial distribution and predictors of postnatal care utilization during the critical time in Ethiopia using EDHS 2019: A spatial and geographical weighted regression analysis

Muluken Chanie Agimas[1], Tigabu Kidie Tesfie[1], Nebiyu Mekonnen Derseh[1], Meron Asmamaw[1], Habtamu Wagnew Abuhay[1], Getaneh Awoke Yismaw[1]

[1]Department of Epidemiology and Biostatistics, institute of public health, college of medicine, university of Gondar, Gondar, Ethiopia.

1. Muluken Chanie Agimas (MCA): mulukensrc12@gmail.com
2. Tigabu Kidie Tesfie (TKT): tigabukidie@gmail.com
3. Nebiyu Mekonnen Derseh (NMD): nebiyumek12@gmail.com
4. Meron Asmamaw (MA): merryalem101@gmail.com
5. Habtamu Wagnew Abuhay (HWA): hab7wagnew@gmail.com
6. Getaneh Awoke Yismaw (GAY): getanehawoke9@gmail.com

**Corresponding author:**

(MCA): mulukensrc12@gmail.com

**To: PLOS ONE <em@editorialmanager.com>**

**Manuscript Number: PONE-D-23-41347R1**

**<u>Cover Letter for round two reviewer's comments and authors responses</u>**

We would like to thank both the Editorial board members and reviewers for kind comments and suggestions. Accordingly, we have tried to correct and respond to those comments point by point, page by page, and line by line. Therefore, we prepared this author's response, the revised manuscript, the separate revised figures and tables, and the tracked changes of the manuscript.

Sincerely, with best regards!

Muluken Chanie Agimas

ID: https://orcid.org/0000-0003-0808-124X

| **Comments from reviewers** | **Authors response** |
| --- | --- |
| The current title of the study is incomprehensible and does not reflect the work. The title of the study must be revised so as to properly reflect the subject matter of the study. | Dear reviewer thank you so much for your advice and valuable comment. Now the title has been revised accordingly. |
| The authors should thoroughly revise the language. The paper is replete with grammatical errors and incoherent sentences. For example, line 93 is incomprehensible and a sentence in line 95 is hanging. It has very basic errors English errors like nonbeliever written as none-believer. | Dear reviewer thank you so much for your recommendation. Now we have revised the grammar of the whole document. |

| | |
|---|---|
| Line 33 “missing data was managed by imputation”, however I could not find any further detail on this. Authors should describe, what was the count of missing values and how it was imputed. | Dear reviewer sorry for the edition problem. The problem happened because we were planning to manage the missing data using the imputation method. But we didn’t face the problem of missing data on the variables we used during the analysis stage. We apologize again for the wrongly written method without revision. Now we have revised this editorial problem accordingly. |
| Introduction section of the study must also briefly engage with the pathways between PNC and health outcomes | Dear reviewer very good question. Dear reviewer, an introduction section should be constructed in a self-informed manner. Based on this, we tried to build the introduction based on the following basic concepts:<br>1. Definition<br>2. Schedule of PNC service<br>3. Health outcome of PNC utilisation (lines 74–78).<br>4. Consequences of Non-Uptake of PNC Utilisation<br>5. Factors for PNC utilisation<br>6. Gaps in the previous research<br>Therefore, we revised the introduction section based on the above important points. |

| | |
|---|---|
| | |
| Critical time is a key-word of the study but I could not find its detailed description/definition/conceptualization in the paper. It must be included. | Dear reviewer thank you for your excellent view and we accept the comment. Necessary amendment has been made. |
| Under the variable religion, does Orthodox mean "Orthodox Christian", please specify it everywhere (page 13). | Dear reviewer you are right orthodox mean orthodox Christian and revision has made on it. |
| The findings section should be revised and reorganized. The section currently is haphazard and complex from the perspective of comprehension. The entire section should be re-written. The authors may also like to organize their figure in a better way, currently there are more than 11 figures and in my understanding many of them can be combined. | Dear Reviewer, we believed that the result section should be organized by the objective of the study. Hence, we try to organize in such a way.<br><br>We try to report the characteristics of the participant at the beginning of the result section, then we report the first objective, which is to report the prevalence of early PNC. Then the next important report and objective was spatial analysis, and we tried to report all the spatial outputs. Then the next step was GWR analysis, and we reported and interpreted accordingly. Dear reviewer, for your respect, we try to review different report styles from the previous articles, and they report or write like what others have reported. If we reduce the content, it will miss important information. To be more attractive and |

| | |
|---|---|
| | readable, we have revised the grammatical issue, re-synthesized the whole section, and reduced the number of figures and tables. If you tell us with specific concern what we are going to revise, we are ready to address the issue. |
| The authors, in conclusion section, conclude that the "government of Ethiopia should increase access to antenatal care", however in the discussion presented by the authors contradicts this. | Dear reviewer our findings showed that the ANC service increases the chance of PNC service. This could be because women with ANC visits have an opportunity to get counselling services about PNC tilization. We try to make recommendations based on this finding. If it causes confusion, we can revise accordingly. |

**Thank you, dear editor and reviewers, for your great scientific contribution!**
