# Supplementary material for: Geospatial distribution and predictors of postnatal care utilization during the critical time in Ethiopia using EDHS 2019: A spatial and geographical weighted regression analysis
Source: PLoS One. 2024 Dec 30;19(12):e0309929. doi: 10.1371/journal.pone.0309929 (PMC11684686; doi:10.1371/journal.pone.0309929)
Supplement: Attachment — Submitted filename: review PNC_EDHS2019.docx [file pone.0309929.s001.docx]

### Dear editors for PLOS ONE

Thank you for allowing me to review this paper. The paper is addressed important public health topic. I have provided some question and comments as outlined below.

**General question**

1. Is it really necessary to conduct additional research when a comparable study was completed in Ethiopia?
2. What will it add to the already known facts?
3. What was the gap in the previous study?
4. Why you select geographical weighted regression analysis for this study?
5. Since EMDHS 2019 the data were not collected all variable like the variables; Media exposure such as (internet use, watching TV, listening radio, and reading newspaper), husband education status, maternal working status and so on. These variables were the most significant factor for postnatal utilization during the critical time and it may affect full research finding. So how to treat those variables in your study?

**General comment**

In abstract section particularly in result and conclusion part, it is too lengthy & try to summarize the sentence and it should incorporate the mothed that you used for spatial analysis.

Study design and setting section you should study area map. Since your study, analysis was spatial regression so it is better to show your study area within a single map

In your Sampling procedure and sampling technique section, “2105 women aged 15-49 years who give birth preceding 2019 EDHS survey were included for the study”. It is weighted sample size or not? It should be clear it maybe weighted or unweighted sample size.

In your method section especially spatial analysis part, you should write all spatial analysis technique separately like spatial auto-correlation, hotspot analysis, and interpolation and SaTScan analysis and cite appropriate citation. In addition, why you select ordinary kriging interpolation technique from other interpolation technique like deterministic like inverse distance weighted(IDW) and geostatistical interpolation methods among those methods simple, ordinary Kriging and universal Kriging?

**Note:** See the results section, especially table 3 for the OLS result. Since your explanatory variables, such as primary education and delivery by CS, were not statically significant variables, your OLS result is unreliable and incorrect. As a result, you should reanalyze your spatial regression by removing those two variables in OLS and GWR analysis and map out all statically significant variables. This breaks the assumption of OLS analysis that violet explanatory variables are statically significant. Rewrite your abstract, results, discussion, and conclusions section overall in light of the updated data.
