# Supplementary material for: Geospatial distribution and predictors of postnatal care utilization during the critical time in Ethiopia using EDHS 2019: A spatial and geographical weighted regression analysis
Source: PLoS One. 2024 Dec 30;19(12):e0309929. doi: 10.1371/journal.pone.0309929 (PMC11684686; doi:10.1371/journal.pone.0309929)
Supplement: Attachment — Submitted filename: Plos one review.docx [file pone.0309929.s003.docx]

1. The current title of the study is incomprehensible and does not reflect the work. The title of the study must be revised so as to properly reflect the subject matter of the study.
2. The authors should thoroughly revise the language. The paper is replete with grammatical errors and incoherent sentences. For example, line 93 is incomprehensible and a sentence in line 95 is hanging. It has very basic errors English errors like nonbeliever written as none-believer.
3. Line 33 “missing data was managed by imputation”, however I could not find any further detail on this. Authors should describe, what was the count of missing values and how it was imputed.
4. Introduction section of the study must also briefly engage with the pathways between PNC and health outcomes.
5. Critical time is a key-word of the study but I could not find its detailed description/definition/conceptualization in the paper. It must be included.
6. Under the variable religion, does Orthodox mean “Orthodox Christian”, please specify it everywhere (page 13).
7. The findings section should be revised and reorganized. The section currently is haphazard and complex from the perspective of comprehension. The entire section should be re-written. The authors may also like to organize their figure in a better way, currently there are more than 11 figures and in my understanding many of them can be combined.
8. The authors, in conclusion section, conclude that the “government of Ethiopia should increase access to antenatal care”, however in the discussion presented by the authors contradicts this.
